# Updates on Potential Therapeutic Approaches for Vitiligo: Janus Kinase Inhibitors and Biologics

**DOI:** 10.3390/jcm12237486

**Published:** 2023-12-04

**Authors:** Valentina Pala, Simone Ribero, Pietro Quaglino, Luca Mastorino

**Affiliations:** Dermatology Clinic, Medical Sciences Department, University of Turin, 10121 Torino, Italy; valentinapala@live.it (V.P.); simone.ribero@unito.it (S.R.); pietro.quaglino@unito.it (P.Q.)

**Keywords:** vitiligo, hypopigmentation, JAK inhibitors, biologics

## Abstract

Vitiligo, the most prevalent skin depigmenting disease, is characterized by the selective loss of melanocytes, impacting patients’ quality of life significantly. This autoimmune disorder progresses through a complex interplay of genetic and non-genetic factors, posing challenges in comprehending its pathogenesis and devising effective treatment strategies for achieving remission. Existing conventional therapeutic approaches, such as topical and oral corticosteroids, calcineurin inhibitors, and phototherapy, lack specificity, offer modest efficacy, and may entail potential adverse effects. Consequently, there is a pressing need for a more nuanced understanding of vitiligo’s pathogenesis to pave the way for targeted therapeutic innovations. This review aims to provide a comprehensive overview of recent developments and findings concerning Januse Kinase (JAK) inhibitors and biologics tested in vitiligo patients. JAK inhibitors have exhibited promising results, showcasing both efficacy and tolerability. In contrast, the outcomes of biologics treatment have been more varied. However, to establish a clearer understanding of which specific pathways to target for a more effective approach to vitiligo, additional in vitro studies and extensive clinical research involving a larger population are imperative.

## 1. Introduction

Vitiligo, an acquired chronic skin disorder, is characterized by patchy depigmentation reflecting the selective loss of melanocytes [1]. Globally, it stands as the leading cause of depigmentation, affecting approximately 1% of the world’s population. While it can manifest at any age, it tends to occur more frequently between 10 and 30 years, with no significant variations in prevalence based on sex, ethnic groups, or skin types [2].

This condition is broadly categorized into two types: nonsegmental vitiligo (NSV) and segmental vitiligo (SV) [3,4]. NSV is marked by depigmented macules of varying size, presenting a progressive course that spreads on both sides of the body. It encompasses acrofacial, mucosal, generalized, and universal subtypes. On the other hand, SV exhibits an earlier onset, rapid development, and is limited in time and space. Depigmentation occurs over a period of 6–24 months, typically following a predominantly unilateral distribution pattern. Mixed vitiligo (MV) is characterized by the coexistence of SV and NSV, often evolving from an initial SV with subsequent development of bilateral NSV patches several months later [5].

Vitiligo’s multifactorial etiology involves factors such as genetic predisposition, oxidative stress due to melanocyte redox balance dysregulation, autoimmunity, and autoinflammatory processes [6,7,8]. Given the complexity of its pathogenesis, managing vitiligo poses a challenge in dermatology. Ideally, treatment should aim to halt the immune system’s impact on melanocytes, stabilize depigmented lesions, and stimulate repigmentation.

Current conventional therapies for vitiligo are limited to generic immunosuppressants, including topical and oral corticosteroids, calcineurin inhibitors, phototherapy, and surgical treatments [9,10]. While narrowband ultraviolet B (NB-UVB) phototherapy is a well-tolerated option that provides a robust stimulus for skin repigmentation by activating melanocyte precursors, its long-term therapeutic effect remains constrained.

Recent research into the molecular mechanisms of this skin disease has unveiled potential targets for treatment, offering the prospect of more specific and effective therapies. This review provides an update on novel molecular targeted therapies for the treatment of vitiligo.

## 2. JAK Inhibitors—Tofacitinib

Janus kinases (JAKs) are a family of intracellular tyrosine kinases composed of four members: JAK1, JAK2, JAK3, and TYK2. The JAK/STAT signaling pathway is involved in modulating immune cell activation and T-cell-mediated inflammation in response to various cytokines. The transmission of signals by IFNγ, a key player in the development of vitiligo, relies on the JAK–STAT pathway. The binding of this cytokine to its specific receptor triggers the activation of the associated JAKs, initiating STAT phosphorylation. Subsequently, the phosphorylated STAT molecules translocate to the nucleus, where they regulate the gene expression of CXCL9 and CXCL10. These chemokines promote the targeted destruction of melanocytes by autoreactive CD8+ T cells, causing skin depigmentation. The return of pigmentation may happen when the inflammatory response is suppressed, suggesting the potential of JAK inhibitors as a new therapy for the treatment of this skin disorder [1] (Table 1). 

Craiglow and colleagues were the first that showed the successful use of tofacitinib in a 50-year-old woman with widespread and progressive vitiligo [11]. Treatment commenced with oral tofacitinib at a dosage of 5 mg every other day, and after 3 weeks, the dosage was escalated to 5 mg daily. Following 2 months of therapy, there was noticeable partial repigmentation on the face and upper extremities. After 5 months, significant repigmentation on the forehead and hands was nearly complete, with the remaining affected areas showing partial repigmentation. Approximately 5% of the total body surface area remained depigmented. The patient exhibited good tolerance to tofacitinib without adverse effects, and laboratory monitoring showed no abnormalities in complete blood cell count, serum creatinine, hepatic function, or lipids throughout the treatment duration.

Song and colleagues evaluated, in a clinical study, the efficacy and safety of tofacitinib combined with NB-UVB phototherapy in 15 refractory nonsegmental vitiligo patients [12]. They observed that the repigmentation level and the response rate were significantly higher in patients treated with tofacitinib 5 mg twice daily + topical drugs and phototherapy compared with the control group (19 patients) treated with topical drugs and phototherapy. Moreover, they reported that no patients treated with tofacitinib developed serious adverse events except for one, and the overall response rate after 16 weeks was 100% in the combination group versus 36.84% in the control group, suggesting that tofacitinib in combination with NB-UVB phototherapy may provide an effective and safe alternative treatment for patients with active/stable nonsegmental refractory vitiligo. 

In a multicenter observational retrospective study, Gianfaldoni et al. evaluated the efficacy and safety of NB-UVB microphototherapy, used alone or in combination with tofacitinib (10 mg/die), in 67 patients with stable or active forms of localized vitiligo [13]. In accordance with a previous study, the group of nine patients treated with tofacitinib showed a better repigmentation rate compared with the control group, without side effects. 

Scheinberg et al. noted substantial improvement in both rheumatoid arthritis and vitiligo in a 30-year-old woman undergoing treatment with tofacitinib at a dosage of 5 mg twice daily. After four months of continuous therapy, there was nearly complete regression of hypopigmentation [14]. Rapid repigmentation was also reported during treatment with tofacitinib + low-dose NB-UVB in two patients with vitiligo with significant facial involvement. These results support the hypothesis that photoactivation is required to stimulate melanocytes, while tofacitinib suppresses the autoimmune response [15]. Liu and colleagues provided retrospective evidence for this hypothesis through a study involving 10 vitiligo patients treated with tofacitinib at a daily dosage ranging from 5 to 10 mg, administered once or twice daily, over an average duration of 9.9 months [16]. Indeed, in the 5/10 patients who achieved a treatment response, repigmentation occurred only in skin areas exposed to the sun or undergoing concomitant NB-UVB phototherapy. Accordingly, Joshipura et al. observed preferential repigmentation over sun-exposed skin area in a 43-year-old female patient with rheumatoid arthritis and generalized vitiligo treated with tofacitinib 5 mg twice daily [17]. 

In contrast to these findings, Fang et al., in a study involving four patients, demonstrated that a combination of low-dose tofacitinib and NB-UVB therapy for 16 weeks proved inadequate for treating refractory vitiligo, with only one out of four patients exhibiting significant repigmentation [18]. Encouragingly, a subsequent study by the same researchers showed more promising results when treating three patients with low-dose tofacitinib in combination with 308 nm excimer light [19]. Furthermore, Komnitski and collaborators detailed a clinical case involving a 40-year-old female patient with rheumatoid arthritis and vitiligo who underwent treatment with tofacitinib at a dosage of 5 mg twice daily for two years, without exposure to ultraviolet light. Following eight months of therapy, notable improvement was observed in macules and patches, marked by the emergence of several repigmented islands on the hands and face. Over the subsequent two years, complete repigmentation was evident on the forehead and perilabial macules, with partial repigmentation occurring in the posterior region of the neck and upper chest. [20]. 

Satisfactory results following oral therapy with tofacitinib were also observed in a 30-year-old patient with concomitant alopecia areata, vitiligo, and plaque and inverse psoriasis and in a 44-year-old white man with atopic dermatitis, alopecia areata, and a 3-year history of nonsegmental multifocal vitiligo [21,22]. 

Tofacitinib efficacy in vitiligo was also evaluated in a topical formulation with the intent to limit systemic side effects [23,24,25,26]. McKesey and colleagues reported a study on 11 patients with vitiligo with significant repigmentation of facial vitiligo lesions after treatment with tofacitinib 2% cream twice daily in combination with NB-UVB therapy for a period of 3 ± 1 months [26]. Excellent results were also reported in young patients with a long history of early-onset generalized, and refractory vitiligo [23,25].

Despite the findings, these studies have several limitations, such as a small sample size, a retrospective design, and the absence of a control group. Subsequent research with larger cohorts and extended follow-up periods is imperative to assess both the efficacy and safety of tofacitinib in the treatment of vitiligo.

## 3. JAK Inhibitors—Ruxolitinib

Ruxolitinib cream is a JAK1 and JAK2 inhibitor that was approved by the FDA after the assessment of its efficacy and safety in many studies [53].

Rosmarin and colleagues reported promising results in adolescents and adults with nonsegmental vitiligo treated with ruxolitinib cream obtained from two phase 3, double-blind, vehicle-controlled studies (Topical ruxolitinib Evaluation in Vitiligo Study 1 (TRuE-V1) and 2 (TRuE-V2)) [27]. In total, 330 and 344 patients were enrolled in these two studies, of whom 221 and 229, respectively, were treated with ruxolitinib 1.5% cream twice daily on all depigmented areas of the face and body. Moreover, they investigated the efficacy of the cream at different doses (1.5% BID, 1.5% QD, 0.5% QD, and 0.15% QD) in treating segmental and nonsegmental vitiligo in a multicenter, randomized, double-blind, vehicle-controlled phase 2 study. The treatment was well tolerated, with the highest response being achieved with a dose of 1.5% BID at 52 weeks, and disease stabilization was associated with a serum CXCL10 reduction, reflecting impaired IFNγ-mediated pathogenesis [28]. 

Rothstein and colleagues examined the efficacy of applying ruxolitinib cream with a concentration of 1.5% twice daily in 11 individuals with vitiligo for a duration of 20 weeks. [29]. They observed that the treatment was more effective for vitiligo located on the face than on other parts of the body. The authors hypothesize that the thinner epidermis and greater exposure to sunlight of the face improve drug absorption compared with the trunk and proximal extremities. In addition, two patients had repigmentation on untreated eyelids, suggesting the possible efficacy of ruxolitinib in counteracting peripheral inflammation when applied to the skin adjacent to the eyelids by allowing periocular repigmentation. After an additional 32 weeks of treatment, no significant improvement was observed in previously unresponsive sites, but a greater response was observed with the combination NB-UVB and ruxolitinib, confirming more pronounced efficacy for facial treatments [30].

In addition, the resistance of acral sites to repigmentation may be due to the lower acral density of pilosebaceous follicles [54]. Camacho et al. reported repigmentation in an 8-year-old male with nonsegmental eyelid vitiligo treated with 1.5% ruxolitinib cream BID for several weeks, without adverse effects [31]. Moreover, following treatment with ruxolitinib 1.5% cream BID, a 49-year-old man suffering for 11 years from generalized vitiligo with approximately 90% facial involvement showed significant repigmentation of the face preferentially in sun-exposed areas and the eyelids, an area where the cream was not applied [17]. Harris and colleagues reported the efficacy of oral ruxolitinib 20 mg BID in a patient with alopecia areata and vitiligo. After 20 weeks, the patient presented with successful repigmentation, particularly in the face, but 12 weeks after treatment discontinuation, while maintaining hair regrowth, much of the regained pigment had receded [32].

## 4. Other JAK Inhibitors in Vitiligo

Baricitinib is a small molecule primarily targeting the JAK1 and JAK2 subtypes and is already utilized in dermatology for inflammatory dermatoses driven by JAK/STAT signals [55]. Mumford et al. initially reported the effectiveness of baricitinib in a 67-year-old Caucasian man with vitiligo on the hands and forearms refractory to tofacitinib treatment [33]. The patient transitioned to baricitinib 4 mg daily and achieved nearly complete repigmentation after 8 months without experiencing adverse effects.

Dong et al. documented the efficacy and tolerability of baricitinib in treating four patients with nonsegmental vitiligo exhibiting progressive disease. However, at the 3-month follow-up, depigmentation occurred in two patients [34]. The authors also investigated the in vitro mechanism of baricitinib in cultures of melanocytes irradiated with high doses of ultraviolet B radiation to simulate damaged melanocytes. They observed that baricitinib could restore tyrosinase activity and melanin synthesis in UV-damaged melanocytes, along with an upregulation of TYR and TRP-1 genes involved in melanogenesis. Two patients with vitiligo showed promising results after combining baricitinib with NB-UVB phototherapy [35].

Upadacitinib, an oral JAK inhibitor selectively inhibiting JAK1 and blocking the signaling of various pro-inflammatory mediators, was studied by Su and colleagues [56]. They assessed the efficacy and safety of upadacitinib 15 mg in vitiligo patients who had not responded to previous treatments with systemic steroids, calcineurin inhibitors, and phototherapy [36]. Facial lesions exhibited greater improvement than acral lesions, and no correlation between repigmentation and sex, age, disease duration, or Fitzpatrick skin type was observed. Pang and colleagues reported the case of a 16-year-old boy with atopic dermatitis since childhood and rapidly progressing vitiligo over the past 4 months. Treatment with upadacitinib led to significant improvement in both conditions, with increased repigmentation in sunlight-exposed areas [37].

In the literature, there is a report detailing the effects of delgocitinib on two patients with vitiligo vulgaris on the neck and elbow, respectively [38]. Delgocitinib is a topical JAK inhibitor that inhibits JAK1, JAK2, JAK3, and tyrosine kinase 2 [57]. Patients receiving delgocitinib treatment twice daily showed a remarkable response on the neck but not on the elbow, possibly due to differences in skin thickness, disease duration, and sun exposure.

## 5. Biologics

Recent studies have provided important insight into the pathogenesis and maintenance of vitiligo, revealing a central role of cell-mediated immunity cytokines in the depigmentation process. 

Abnormal immune responses and changes in cytokine levels observed in vitiligo patients suggest that targeting pro-inflammatory mediators including TNFα and ILs-12, 23, and −17 could be a promising treatment strategy [58,59]. Nevertheless, cases of vitiligo induced by biological drugs are reported in the literature [39,60]. Burlando’s group reported a case of a 32-year-old male with worsening psoriasis and stable vitiligo patches in the subaxillary and perioral area. Following treatment with ustekinumab, a monoclonal antibody that targets both IL-12 and IL-23, the psoriasis improved but a new hypopigmented lesion appeared close to the resolved psoriasis patches, which further extended in the following months, while the pre-existing patches remained stable [39]. In contrast, Elkady and colleagues reported successful results on vitiligo patches in a patient with concomitant psoriasis, vitiligo, and alopecia areata [40]. Alghamdi’s group investigated the efficacy and safety of anti-TNFα agents in six vitiligo patients (two patients treated with infliximab, two with adalimumab, and two with etanercept). No patient showed repigmentation following treatment, and worsening was observed in one patient treated with infliximab [41]. The lack of efficacy of anti-TNFα treatment was reported in other studies [42,46]. On the other hand, Kim’s group observed improvement in refractory vitiligo in two patients treated with the anti-TNFα agent etanercept [43]. They also analyzed and correlated tissue levels of TNFα with disease activity of vitiligo, pointing to TNFα as a potential predictive biomarker of the response to anti-TNFα treatment in refractory nonsegmental vitiligo. 

Campanati et al. also described mild improvement in vitiligo lesions during etanercept treatment [44]. Moreover, a 24-year-old male with ankylosing spondylitis and generalized vitiligo showed significant repigmentation after 6 months of infliximab treatment [47]. 

Controversial results were also reported in vitiligo patients treated with the anti-IL17 agent secukinumab. A 63-year-old man with psoriasis who developed vitiligo during therapy with adalimumab showed near complete repigmentation after one year of secukinumab administration [48]. Moreover, a 1-year-old boy with generalized pustular psoriasis developed generalized vitiligo after three weeks of acitretin treatment that improved with secukinumab in combination with topical steroid [49]. Gradual repigmentation of the face and trunk was observed from the fifth and twelfth administration of secukinumab, respectively, while no significant improvement was observed on the scalp. In contrast, in a single-arm pilot study with active nonsegmental vitiligo patients treated with secukinumab, seven out of eight patients developed additional skin depigmentation [50]. 

Moreover, Argüelles and colleagues reported mixed results for active disseminated vitiligo of five patients treated with a single intravenous infusion of rituximab, a monoclonal antibody to CD20 [51]. During the 6-month follow-up, improvement in vitiligo in four patients was observed, while one patient presented no change. 

A 63-year-old man with progressive vitiligo showed 90% repigmentation of acrofacial vitiligo following one year of treatment with tildrakizumab, an anti-IL23 monoclonal antibody [52].

## 6. Discussion

Vitiligo is the most common depigmenting disorder that negatively influences patients’ quality of life and causes significant psychological distress [2,61,62,63]. Current treatments for vitiligo include topic and oral corticosteroids, phototherapy, and calcineurin inhibitors that are nonspecific and exhibit short-term efficacy and many potential side effects.

**Figure 1:**
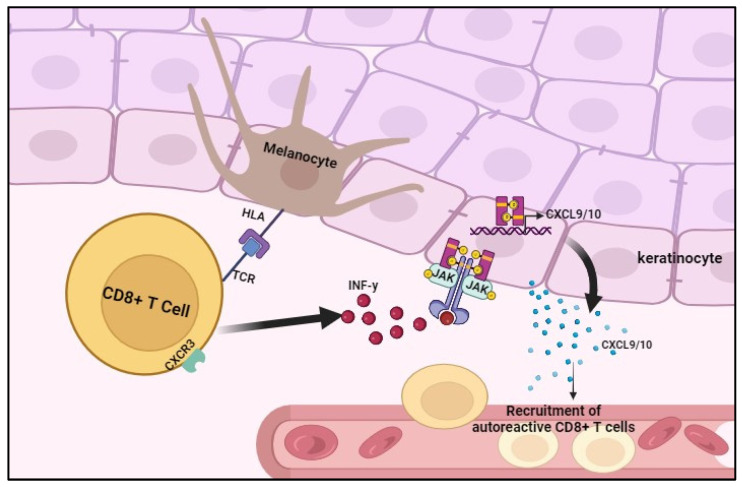
Recruitment of CD8+ T cells during progression of vitiligo.

Vitiligo is an immune-mediated disease characterized by a complex inflammatory cascade initiated by innate immune cells that recognize signals from stressed melanocytes [7,64]. Upon activation, innate immune cells produce IFN-γ, which stimulates secretion of CXCL9 and CXCL10 by keratinocytes through activation of the JAK/STAT signaling pathway, resulting in the recruitment of CD8+ T cells through the CXCR3 receptor. Vitiligo progression is characterized by a positive feedback loop that promotes continuous T-cell recruitment through IFN-γ secreted by autoreactive CD8+ T cells and activation of the JAK/STAT signaling pathway in keratinocytes (Figure 1). 

With recent advances in our understanding of the molecular mechanisms driving the pathogenesis of vitiligo, JAK inhibitors are emerging as promising therapeutic options. Moreover, in a case–control study, skin biopsies of 24 patients with active vitiligo and 20 healthy volunteers were analyzed to evaluate the expression of JAK1, 2, and 3 using RT-PCR. JAK1 and particularly JAK3 showed overexpression in the skin of vitiligo patients compared with the controls, with a gradual shift from nonlesional to perilesional and lesional sections, with no correlation between the expression levels of JAK1 and JAK3 and disease activity or severity. In contrast, JAK2 expression showed no significant differences compared with control skin [65].

Nowadays, data in the literature report promising results and good tolerability of JAK inhibitors and conflicting results for biological drugs. 

Given the expansive array of therapeutic options discussed in the literature for treatment selection, it is imperative to factor in the safety profile of the chosen drug [66]. In this context, it becomes pertinent to note that topical treatments, in comparison with systemic treatments, have fewer demonstrated adverse events and contraindications. However, it is essential to acknowledge that their applicability is frequently confined to localized diseases. 

Moreover, other biologics and JAK inhibitors for vitiligo treatment are currently under investigation in clinical trials (Table 2). 

## 7. Limitations

While JAK inhibitors, specifically tofacitinib and ruxolitinib, have shown promising results, it is essential to conduct additional studies involving a larger population and extended follow-up periods. These investigations are necessary to comprehensively assess the long-term efficacy and safety of these therapeutic agents. Additionally, it is important to deeply investigate the role of cytokines such as TNFa, IL17, and IL23 in the pathogenesis of vitiligo in order to elucidate the potential utility of biologics in vitiligo treatment. 

## 8. Conclusions

Numerous small-scale studies and case reports have demonstrated favorable outcomes with JAK inhibitors for vitiligo treatment, utilizing both topical and oral administration, frequently in conjunction with phototherapy. In contrast, the literature concerning the therapeutic impact of biologics on vitiligo is restricted and inconclusive, with success documented in only a few cases.

## Figures and Tables

**Table 1:** Novel potential JAK inhibitors and biologics in vitiligo.

Treatment	N° Patients	Type of Vitiligo	Administration	Combined Treatment	Study Type	Duration	Adverse Events	References
Tofacitinib	A 50-year-old female	Not specified	Oral 5 mg every other day. After 3 weeks, 5 mg QD	No	Case report	5 months	No	[11]
Tofacitinib	42	Nonsegmental vitiligo	Oral tofacitinib 5 mg BID	Halometasone cream + tacrolimus 0.1% ointment or pimecrolimus cream + NB-UVB therapy	Clinical study	16 weeks	1 mild pain, abnormal levels of blood lipids, uric acid, and coagulation function	[12]
Tofacitinib	9	Vitiligo vulgaris	Oral tofacitinib 10 mg	micro-focused NB-UVB phototherapy	Multicenter observational retrospective study	36 weeks	No	[13]
Tofacitinib	A 30-year-old female	Not specified	Oral tofacitinib 5 mg BID	No	Case report	16 weeks	Not reported	[14]
Tofacitinib	A 50-year-old man + a 30-year-old woman	Not specified	Oral tofacitinib 5 mg BID	NB-UVB phototherapy	Case report	6 months + 3 months	No	[15]
Tofacitinib	10	8 generalized vitiligo + 2 acral vitiligo	Oral tofacitinib 5–10 mg QD-BID	Alone or in combination with NB-UVB phototherapy	Retrospective case series	3–15 months	2 upper respiratory infection, 1 weight gain, 1 arthralgias, and 4 mild elevation in lipids	[16]
Tofacitinib	A 43-year-old female	Generalized vitiligo	Oral tofacitinib 5 mg BID	No	Case report	12 weeks	Not reported	[17]
Tofacitinib	4	Not specified	Oral tofacitinib 5 mg QD	NB-UVB phototherapy	Pilot study	16 weeks	No serious adverse events	[18]
Tofacitinib	3	Nonsegmental vitiligo	Oral tofacitinib 5 mg QD	308 nm excimer light	Clinical study	12 weeks	No severe adverse events	[19]
Tofacitinib	A 40-year-old female	Not specified	Oral tofacitinib 5 mg BID	No	Case report	2 years	Not reported	[20]
Tofacitinib	A 30-year-old male	Nonsegmental vitiligo	Oral tofacitinib 5 mg BID. After 4 months, 5 mg QD	NB-UVB phototherapy	Case report	More than 1 year	Headache and flu-like symptoms	[21]
Tofacitinib	A 44-year-old male	Nonsegmental multifocal vitiligo	Oral tofacitinib 5 mg BID	Prednisolone 5 mg QD and betamethasone dipropionate ointment	Case report	6 months	Upper respiratory infections and diarrhea	[22]
Tofacitinib	A 17-year-old boy	Nonsegmental vitiligo with acrofacial involvement	Topical tofacitinib 2% BID	NB-UVB phototherapy	Case report	9 months	Erythema and transient acne	[23]
Tofacitinib	16	Nonsegmental vitiligo	Topical tofacitinib 2% BID	Topical steroids, topical calcineurin inhibitors, supplements, or phototherapy	Pilot study	Not specified	1 acne-like papules and 1 subtle skin contour changes	[24]
Tofacitinib	A four-year-old boy	Segmental vitiligo	Topical tofacitinib 2% BID	NB-UVB phototherapy	Case report	6 months	No	[25]
Tofacitinib	11	Facial vitiligo	Topical tofacitinib 2% BID	NB-UVB phototherapy	Pilot study	3 months ± 1	Not reported	[26]
Ruxolitinib	A 49-year-old male	Generalized vitiligo	Topical ruxolitinib 1.5% BID	No	Case report	38 weeks	Not reported	[17]
Ruxolitinib	221	Nonsegmental vitiligo	Topical ruxolitinib 1.5% BID	No	Double-blind, vehicle-controlled trials: TRuE-V1	52 weeks	Application-site acne, pruritus, and exfoliation anal fistula, appendicitis, concussion, hepatitis due to infectious mononucleosis, hypersensitivity, kidney contusion, myocarditis, prostate cancer, and subacute combined cord degeneration	[27]
Ruxolitinib	228	Nonsegmental vitiligo	Topical ruxolitinib 1.5% BID	No	Double-blind, vehicle-controlled trials: TRuE-V2	52 weeks	Application-site acne, pruritus, and exfoliation, appendiceal abscess, coronary artery stenosis, joint dislocation, papillary thyroid cancer, rhabdomyolysis, and ureterolithiasis	[27]
Ruxolitinib	125	6 segmental + 119 nonsegmental vitiligo	Topical 1·5% BID, 1·5% QD, 0·5% BID, 0·15% QD	No	Randomized, double-blind, dose-ranging, phase 2 trial study	52 weeks	Application-site acne and pruritus, pruritus, and headache	[28]
Ruxolitinib	11	Nonsegmental vitiligo	Topical ruxolitinib 1.5% BID	No	Open-label, proof-of-concept trial	20 weeks	Erythema, hyperpigmentation around vitiligo patches, and papular eruptions or worsening of acne	[29]
Ruxolitinib	8	Nonsegmental vitiligo	Topical ruxolitinib 1.5% BID	Optional NB-UVB phototherapy	Open-label extension study	52 weeks	Minor adverse events, including erythema and transient acne	[30]
Ruxolitinib	8-year-old male	Nonsegmental eyelid vitiligo	Topical ruxolitinib 1.5% BID	No	Case report	Not specified	No	[31]
Ruxolitinib	A 35-year-old male	Not specified	Oral ruxolitinib 20 mg BID	No	Case report	20 weeks	Not reported	[32]
Baricitinib	a 67-year-old male	Vitiligo in hands and forearms	Oral baricitinib 4 mg QD	No	Case report	8 months	No	[33]
Baricitinib	4	Nonsegmental vitiligo	Oral baricitinib 4 mg QD for 4 weeks, 2 mg QD for 8 weeks	No	Clinical study	12 weeks	No	[34]
Baricitinib	A 17-year-old woman + 56-year-old woman	Generalized vitiligo	Oral baricitinib 2 mg BID	0.1% tacrolimus ointment + NB-UVB phototherapy and diprospan injection + oral ginkgo biloba 80 mg + topical 0.1% tacrolimus ointment + mometasone furoate cream + NB-UVB phototherapy	Case report	8 months and 6 months	Not reported	[35]
Upadacitinib	12	Not specified	Oral upadacitinib 15 mg QD	No	Clinical study	16–40 weeks	Acne	[36]
Upadacitinib	A 16-year-old boy	Not specified	Oral upadacitinib 15 mg QD	Crisaborole	Case report	16 weeks	Worsened acne	[37]
Delgocitinib	A 39-year-old man + a 45-year-old woman	Vitiligo vulgaris	Topical delgocitinib BID	No	Case report	8 weeks and 12 weeks	No	[38]
Ustekinumab	A 32-year-old male	Not specified	Subcutaneous ustekinumab 90 mg week 0–4, then every 12 weeks	No	Case report	12 months	New vitiligo patch	[39]
Ustekinumab	A 39-year-old female	Nonsegmental vitiligo	Subcutaneous ustekinumab 90 mg week 0–4, then every 8 weeks	No	Case report	16 weeks	No	[40]
Etanercept	A 38-year-old man + a 15-year-old woman	Nonsegmental vitiligo	50 mg subcutaneous injection twice weekly	No	Pilot study	24 weeks	No	[41]
Etanercept	4 males	Not specified	50 mg subcutaneous injection once weekly, followed by 25 mg once week	No	Open-label pilot study	12 weeks + 4 weeks	No	[42]
Etanercept	A 42-year-old woman + a 46-year-old woman	Nonsegmental vitiligo	50 mg subcutaneous injection twice weekly	NB-UVB phototherapy and topical calcineurin	Case report	12 months	Not reported	[43]
Etanercept	A 65-year-old male	Not specified	50 mg subcutaneous injection twice weekly followed by 25 mg twice a week	No	Case report	12 weeks + 12 weeks	No	[44]
Etanercept	A 63-year-old male	Focal vitiligo	50 mg subcutaneous injection twice weekly followed by 25 mg twice a week	Isoniazid	Case report	12 weeks + 12 weeks	No	[45]
Adalimumab	A 31-year-old man + a 24-year-old man	Nonsegmental vitiligo	80 mg subcutaneous injection week 0, from week 1—40 mg subcutaneous injection every two weeks	No	Pilot study	24 weeks	No	[41]
Infliximab	An 18-year-old man + a 22-year-old man	Nonsegmental vitiligo	5 mg/kg intravenous at 0, 2, and 6 weeks, then 5 mg/kg every 8 weeks	No	Pilot study	30 weeks	No	[41]
Infliximab	A 17-year-old male	Vitiligo vulgaris	5 mg/kg intravenous at 0, 2, and 6 weeks, then 5 mg/kg every 6 weeks	No	Case report	24 weeks	Psoriasiform dermatitis	[46]
Infliximab	A 24-year-old male	Generalized vitiligo	5 mg/kg intravenous at 0, 2, and 6 weeks, then 5 mg/kg every 8 weeks	No	Case report	10 months	Not reported	[47]
Secukinumab	A 63-year-old male	Not specified	Subcutaneous 300 mg weekly for 4 weeks, then 300 mg monthly	No	Case report	12 months	No	[48]
Secukinumab	A 1-year-old boy	Segmental vitiligo	Subcutaneous 75 mg weekly for 4 weeks, then 75 mg monthly	Topical steroids	Case report	9 months	Not reported	[49]
Secukinumab	8	Nonsegmental vitiligo	Subcutaneous 300 mg weekly for 4 weeks, then 300 mg monthly	No	A single-arm pilot study	7 months	No	[50]
Rituximab	5	Disseminated active vitiligo	Two 500 mg intravenous infusions 2 weeks apart from each other	No	Pilot study	NA	Not reported	[51]
Tildrakizumab	A 63-year-old man	Acrofacial vitiligo	100 mg subcutaneous injection at weeks zero, four, and twelve	No	Case report	12 months	No	[52]

**Table 2:** Clinical trials for further biologics and JAK inhibitors for vitiligo.

ClinicalTrials.gov ID	Study Phase	Type of Vitiligo	Agent	Classification
NCT05917561	II	nonsegmental progressive vitiligo	Anifrolumab	anti-IFN-α monoclonal antibodies
NCT04338581	IIA	active or stable vitiligo	AMG 714	anti-IL-15 monoclonal antibody
NCT04103060	II	nonsegmental vitiligo	Cerdulatinib	SYK/JAK inhibitor
NCT03468855	II	nonsegmental facial vitiligo	Ifidancitinib (ATI-50002)	JAK1 and JAK3 inhibitor
NCT02281058	I	active vitiligo	Abatacept	fusion protein (CTLA4-Ig)
NCT04818346	II	nonsegmental vitiligo	INCB054707	JAK1 inhibitor
NCT05583526	III	nonsegmental vitiligo	Ritlecitinib	JAK3 and the TEC kinase family inhibitor
NCT03715829	IIb	active nonsegmental vitiligo	Ritlecitinib (PF-06651600), Brepocitinib (PF-06700841)	JAK3 and the TEC kinase family inhibitor, JAK1, and TYK2 inhibitor

## Data Availability

Data available upon reasonable request.
